# Influence of discontinuation of prophylactic antimicrobial agent for trabeculectomy

**DOI:** 10.1186/s40780-023-00276-z

**Published:** 2023-03-01

**Authors:** Yuuka Ushio, Hiroshi Yoshikawa, Tetsuya Murase, Tatsuo Kataoka, Shohei Miyamoto, Kazunari Maruko, Shoko Okamoto, Yuuka Shibata, Ryotaro Toda, Yoshiaki Kiuchi, Hiroaki Matsuo

**Affiliations:** 1 Department of Pharmaceutical Services, Hiroshima University Hospital, 1-2-3, Kasumi, Minami-ku, Hiroshima-shi, Hiroshima, 734-8551 Japan; 2 Department of Ophthalmology and Visual Science, Graduate School of Biomedical and Health Sciences, Hiroshima University, Hiroshima, Japan

**Keywords:** Antibiotic prophylaxis, Endophthalmitis, Trabeculectomy

## Abstract

**Background:**

There is no unified view of the necessity of prophylactic antimicrobial agents in trabeculectomy. Preoperative prophylactic antimicrobial agent injection and cefazolin sodium (CEZ) for trabeculectomy were discontinued at the Hiroshima University Hospital. In this study, we evaluated whether discontinuation of preoperative administration of CEZ in ophthalmology affects the incidence of postoperative infections.

**Methods:**

We retrospectively investigated patient background, concomitant medications, subconjunctival dexamethasone sodium phosphate (DEX) injection at the end of the surgery, and the incidence of infective endophthalmitis within 6 weeks after surgery in the CEZ and non-CEZ groups. We also performed propensity score matching for background matching. Statistical analysis was performed using the Mann-Whitney *U*-test and Fisher’s exact test.

**Results:**

The incidence of postoperative endophthalmitis was not significantly different between 629 and 751 patients in the CEZ and no-CEZ groups, respectively (0 in the CEZ group and 2 in the no-CEZ group, *P* = 0.504). More patients in the CEZ group were taking diabetes drugs preoperatively (*P* = 0.028) and fewer patients were receiving subconjunctival DEX at the end of surgery (*P* < 0.001) than those in the non-CEZ group. Propensity scores were calculated using the risk factors for postoperative infection as covariates, and matching (580 patients in the CEZ group and 580 patients in the non-CEZ group) showed no significant difference in the incidence of postoperative endophthalmitis (*P* = 0.500).

**Conclusions:**

There was no significant difference in the incidence of endophthalmitis after trabeculectomy between the CEZ and non-CEZ groups, suggesting a decreased need for CEZ injections before trabeculectomy.

## Background

Endophthalmitis is a common surgical site infection after trabeculectomy, with acute cases occurring within 6 weeks after surgery. Although the incidence is low, it is a serious complication that can lead to severe vision loss and blindness [1].

Since postoperative endophthalmitis is caused by bacteria indigenous to the conjunctiva and eyelid, antimicrobial agents and povidone-iodine disinfection are generally used to prevent endophthalmitis [2]. In the United States, preoperative administration of antimicrobial eye drops and subconjunctival administration of antimicrobial agents at the end of surgery is recommended to prevent postoperative endophthalmitis [2]. However, most of the literature on which these guidelines are based refers to cataract surgery, and there is no unified view of prophylactic antimicrobial agents in trabeculectomy. This is because the number of glaucoma surgeries is much lower than the number of cataract surgeries. Similarly, Japanese prophylactic antimicrobial guidelines recommend the preoperative use of antimicrobial eye drops for cataract surgery, while intravenous cefazolin sodium (CEZ) is recommended for trabeculectomy with a low level of evidence [3]. Since there have been few studies on prophylactic antimicrobial agents in trabeculectomy [4], no clear evidence of its usefulness has been obtained.

Hiroshima University Hospital performs approximately 600 trabeculectomies per year, ranked second in Japan in terms of the number of surgeries in the Diagnosis Procedure Combination database in the fiscal year 2018 [5]. In addition, CEZ was administered prophylactically before trabeculectomy as a clinical pathway. However, there is no clear evidence that CEZ injections prevent postoperative endophthalmitis. There is also the risk of administering unnecessary antimicrobials, which may cause minor side effects, such as diarrhea, and serious side effects, such as anaphylactic shock with dyspnea. In addition, the “Action Plan for Antimicrobial Resistance Control [6]“has been formulated as a national policy, and it is important to discontinue unnecessary antimicrobial agents based on the viewpoint of controlling drug-resistant bacteria. Therefore, in April 2018, our hospital reviewed the clinical pathway for trabeculectomy and discontinued CEZ injection. Perioperative infection prophylaxis consisted only of povidone-iodine disinfection and antimicrobial eye drops. In this study, we compared the incidence of postoperative endophthalmitis before and after clinical pathway change to determine the impact of discontinuation of prophylactic CEZ injection for trabeculectomy on the incidence of surgical site infection.

## Methods

### Patients

A summary of our trabeculectomy clinical pathway and antimicrobials used is presented in Table 1. We removed the CEZ injection from our clinical pathway starting on April 12, 2018. Patients who underwent trabeculectomy at our hospital on admission were included in this study. The patients were classified into two groups: the CEZ group before the clinical pathway change (February 2, 2016, to April 11, 2018) and the non-CEZ group after the clinical pathway change (April 12, 2018, to December 31, 2020). We excluded patients who were taking oral antimicrobials at admission, who had undergone concomitant procedures other than trabeculectomy, and who were difficult to follow up for 6 weeks postoperatively after trabeculectomy.

**Table 1:** Summary of the trabeculectomy clinical pathway and antimicrobials used

Three days before surgery	Levofloxacin 1.5% eye drops Three times a day
Before surgery	CEZ injection 1 g Disinfection with iodine and polyvinyl alcohol 6x diluted solution (0.033% effective iodine)
During surgery	Application of mitomycin C to the surgical wound ^a)^ (Subconjunctival administration of DEX injection ^a)^)
After surgery	Ofloxacin 0.3% eye ointment
Day after surgery ~ 1–3 months	Levofloxacin 1.5% eye drops Fluorometholone 0.1% eye drops Nepafenac 0.1% suspension eye drops Three times a day

^a)^ Not covered by insurance

### Patient background and primary end point

Patient characteristics included age, sex, obesity (body mass index [BMI] ≥25 kg/m^2^), and concomitant medications. To adjust for factors that might affect the incidence of postoperative endophthalmitis, a propensity score was calculated using patient background parameters as covariates for matching (caliper coefficient:0.2). As it has been reported that age > 85 years is a risk factor for postoperative endophthalmitis in cataract surgery [7], we checked the number of patients in each age group. Concomitant medications known to affect surgical site infection, such as diabetes drugs, immunosuppressants, and corticosteroids (eye drops, eye ointment, oral administration, and subconjunctival dexamethasone sodium phosphate (DEX) injection at the end of surgery), were collected from medical records. Subconjunctival DEX was administered at the discretion of the primary surgeon for anti-inflammatory purposes. Immunosuppressants and corticosteroids were defined based on the efficacy classification of the Japanese Standard Commodity Classification [8].

Patients with postoperative endophthalmitis were defined as those diagnosed with postoperative endophthalmitis by an ophthalmologist within 6 weeks after surgery.

### Statistical analysis

The Mann-Whitney *U*-test was used for age, and the chi-square test was used for sex, obesity, the presence of subconjunctival DEX at the end of the surgery, and concomitant medications other than immunosuppressants. The Fisher’s exact test was used to assess immunosuppressant usage and presence of postoperative endophthalmitis. Using propensity score matching methods, we calculated the propensity scores for CEZ injection using a logistic regression model that included the following variables: age, patients aged 85 years or older, obesity, concomitant medications, and subconjunctival DEX injection. One-to-one nearest-neighbor matching without replacement was performed for the estimated propensity scores in the patients using a caliper width set at 20% of the pooled standard deviation of the logit of the propensity score. A significance level of less than 5% was considered statistically significant. EZR Ver. 1.55 [9] was used for the statistical analysis.

## Results

### Patient background

Of the 889 patients in the CEZ group, 15 taking oral antimicrobial agents on admission and 245 who had undergone procedures other than trabeculectomy were excluded, and 629 patients were included in the analysis. Of the 1063 patients in the non-CEZ group, 15 who were taking oral antimicrobial agents on admission and 297 who had undergone procedures other than trabeculectomy were excluded, and 751 patients were included in the analysis. In both groups, there were no patients with difficulty following up for 6 weeks postoperatively. The patient backgrounds of the two groups are shown in Table 2. There were no significant differences in sex, median age, or obesity (BMI ≥25) between the two groups. There were statistically significant differences between the two groups in terms of subconjunctival DEX at the end of surgery and diabetes drugs. Subconjunctival DEX was administered more frequently at the end of surgery in the non-CEZ group (161 patients [21.4%]) than in the CEZ group (11 patients [1.7%]). The use of diabetes drugs was less common in the non-CEZ group (134 patients (17.8%)) than in the CEZ group (143 patients (22.7%)). There were no significant differences in other concomitant medications between the two groups.

**Table 2:** Patient background

		CEZ (*n* = 629)		Non-CEZ (*n* = 751)		*P*-value
Sex (Male / Female)		340 / 289		426 / 325		0.347^a)^
Age (years)	Median	72		70		0.106^b)^
	Quartile range	64–78		62–78		
Patients 85 years and older		65	(10.3%)	66	(8.8%)	0.377^a)^
Obesity (BMI ≥ 25)		194	(30.8%)	213	(28.4%)	0.344^a)^
Concomitant medications						
Diabetes drugs		143	(22.7%)	134	(17.8%)	^*^0.028^a)^
Immunosuppressant		3	(0.5%)	9	(1.2%)	0.244^c)^
Oral corticosteroids		31	(4.9%)	24	(3.2%)	0.133^a)^
Corticosteroid eye drops and ointment		58	(9.2%)	71	(9.5%)	0.956^a)^
Subconjunctival DEX injection		11	(1.7%)	161	(21.4%)	^*^ < 0.001^a)^

^a)^ Chi-square test

^b)^ Mann-Whitney U test

^c)^ Fisher’s exact test, *P < 0.05

Since the rate of subconjunctival DEX injection at the end of surgery and diabetes drugs differed between the two groups and their possible influence on postoperative infection could be ruled out, propensity score matching was performed to reduce bias. After propensity score matching, 580 patients in the CEZ group and 580 patients in the non-CEZ group were included in the study. The patient backgrounds of the two groups are shown in Table 3. There were no significant differences between the two groups in any of the items.

**Table 3:** Patient background after matching

		CEZ (n = 580)		Non-CEZ (*n* = 580)		*P*-value
Sex (Male / Female)		328 / 252		330 / 250		0.906^a)^
Age (years)	Median	72		70		0.319^b)^
	Quartile range	(63–78)		(63–78)		
Patients 85 years and older		58	(10.0%)	53	(9.1%)	0.690^a)^
Obesity (BMI ≥ 25)		163	(28.1%)	167	(28.8%)	0.845^a)^
Concomitant medications						
Diabetes drugs		120	(20.7%)	118	(20.3%)	0.942^a)^
Immunosuppressant		3	(0.5%)	5	(0.9%)	0.726^c)^
Oral corticosteroids		23	(4.0%)	21	(3.6%)	0.878^a)^
Corticosteroid eye drops and ointment		53	(9.1%)	55	(9.5%)	0.920^a)^
Subconjunctival DEX injection		11	(1.9%)	9	(1.6%)	0.822^a)^

^a)^Chi-square test

^b)^ Mann-Whitney U test, and

^c)^ Fisher’s exact test

### Incidence of postoperative Endophthalmitis

The number of patients diagnosed with postoperative endophthalmitis was 0 (0.0%) in the CEZ group and 2 (0.3%) in the non-CEZ group, with no significant difference (*P* = 0.504) (Table 4). The number of patients diagnosed with postoperative endophthalmitis after matching was 0 (0.0%) in the CEZ group and 2 (0.3%) in the non-CEZ group, with no significant difference (*P* = 0.500) (Table 4).

**Table 4:** Incidence of postoperative endophthalmitis

	All cases			After matching		
	CEZ	Non-CEZ	*P*-value	CEZ	Non-CEZ	*P*-value
Postoperative endophthalmitis	0 / 629 (0.0%)	2 / 751 (0.3%)	0.504	0 / 580 (0.0%)	2 / 580 (0.3%)	0.500

Fisher’s exact test

The background and symptom course of the patients diagnosed with postoperative endophthalmitis are shown in Table 5. None of the patients was aged ≥85 years. In one patient, *Enterococcus faecalis* was detected in the anterior chamber aqueous humor, while in the other patient, no bacteria were detected in either the anterior chamber aqueous humor or vitreous humor. All patients showed improvement in visual acuity after the vitrectomy.

**Table 5:** Postoperative endophthalmitis cases

Case 1	Female in her 80s, Obesity 1st degree, Diabetes drug user [Treatment] Day after surgery: Vitrectomy and intravitreal administration of vancomycin and ceftazidime [Bacteria detection] Anterior chamber aqueous humor: *Enterococcus faecalis*, Vitreous humor (−) [Visual acuity] Before surgery 50 cm/m.m., Day after surgery sl (+), 3 months after 30 cm/m.m.
Case 2	Male in his 70s, Obesity 1st degree [Treatment] 16 days after surgery: Vitrectomy and intravitreal administration of vancomycin [Bacteria detection] Anterior chamber aqueous humor (−), Vitreous humor (−) [Visual acuity] Before surgery 0.3 (0.5 × S), 16 days after surgery 0.01 (0.3 × S), 2 months after 0.3 (0.5 × S)

## Discussion

This report investigated the necessity of administering injectable antimicrobials for the prophylaxis of postoperative endophthalmitis following trabeculectomy. Although there were differences in the use of subconjunctival DEX and diabetes drugs between the CEZ and non-CEZ groups, there were no significant differences in the number of cases of postoperative endophthalmitis between these groups after adjusting for patient background using the propensity score matching method. This suggests that discontinuation of the CEZ injection prior to trabeculectomy does not increase the incidence of postoperative endophthalmitis. There was no change in the clinical pathway other than administering antimicrobials. In addition, the 10-fold increase in the number of subconjunctival DEX injection in the non-CEZ group remains unclear.

The reason for the lack of an increase in the incidence of endophthalmitis after discontinuation of CEZ injection may be that the risk of infection was controlled by povidone-iodine disinfection and antimicrobial eye drops. In addition, the *Enterococcus species* detected in the patient diagnosed with postoperative endophthalmitis in this study were resistant to cephem antimicrobials, suggesting that the use of preoperative CEZ injection was not feasible for prevention in this case.

However, in our hospital, quinolone antibacterial eye drops have been used continuously for 1–3 months postoperatively, but reports of cataract surgery at other hospitals indicate that the administration of quinolone antibacterial eye drops is terminated 3–7 days after surgery [2]. There are no reports investigating the necessity and appropriate duration of eye drop administration, and further studies are required.

This study has several limitations. First, although the risk of surgical site infection associated with oral corticosteroids is related to the dosage and duration of administration [10], it is difficult to evaluate the history of corticosteroid administration in a retrospective study. Second, although poor postoperative glycemic control is considered a risk factor for surgical site infection in patients with or without diabetes mellitus [11], perioperative blood glucose monitoring was not performed in many patients, and as an alternative, the use of diabetes drugs was compared according to the patient background. Finally, the incidence of postoperative endophthalmitis before and after discontinuation of CEZ injections should be compared in a non-inferiority study. However, because the incidence of endophthalmitis after trabeculectomy is very low, a large number of cases are required, which is difficult with the present number of cases. Therefore, we compared all the patients before and after discontinuation using the Fisher’s exact test, which can verify the null hypothesis of independence even when the expected value is small. The number of cases and data analyses at a single institution are limited, and future multicenter data collection and analysis are desirable.

## Conclusion

These results suggest that preoperative CEZ injection is not necessary after trabeculectomy, and that prevention of postoperative endophthalmitis can be achieved with iodine disinfection and antimicrobial eye drops. Further large-scale studies are needed to examine the effects of corticosteroid administration and blood glucose control.

## Data Availability

The data used in this report will not be shared because of the risk of identifying patients’ identities. The most relevant data are presented here.

## Abbreviations

CEZ: Cefazolin sodium
DEX: Dexamethasone sodium phosphate
BMI: Body mass index
